# Factors relating to climate change anxiety in women at reproductive age: A CHAID analysis

**DOI:** 10.1590/1980-220X-REEUSP-2025-0182en

**Published:** 2025-09-08

**Authors:** Tuğçe Sönmez, Enes Karaman

**Affiliations:** 1 Tarsus University, Faculty of Health Sciences, Department of Midwifery, Mersin, Türkiye.; 2 Niğde Ömer Halisdemir University, Faculty of Medicine, Department of Obstetrics and Gynaecology, Niğde, Türkiye.

**Keywords:** Anxiety, Climate change, Midwifery, Reproduction, Women's health, Ansiedade, Mudanças climáticas, Reprodução, Saúde da mulher, Tocologia

## Abstract

**Objective:**

This study aimed to determine the factors relating to climate change anxiety in women at reproductive age.

**Method:**

This study is descriptive and cross-sectional. This study included 351 women aged 18–45 years. Data were collected via face to face interviews using the ‘Women's Descriptive Characteristics and Climate Change Perception Assessment Form’ and ‘Climate Change Anxiety Scale for Women's Health’ between June and October 2024 at a family health center in Mersin province of Türkiye. Data analysis included descriptive statistical methods and chi-squared automatic ınteraction detector analysis.

**Results:**

The average age of the women was 27.91 ± 8.30 years (n = 351). The Climate Change Anxiety Scale for Women's Health mean score was 52.46 ± 17.19. Women's experience of anxiety regarding the negative consequences of climate change affected the scale and all sub-dimensions (physiological health, behavior, and gender) (p < 0.05).

**Conclusion:**

Health professionals should provide information and assessments including the effects of climate change on health in care services. They should plan interventions to reduce women's anxiety levels towards climate change.

## RESUMO

**Objetivo:**

Este estudo teve como objetivo determinar os factores relacionados com a ansiedade face às alterações climáticas em mulheres em idade reprodutiva.

**Método:**

Este estudo é descritivo e transversal. Este estudo incluiu 351 mulheres com idades compreendidas entre os 18 e os 45 anos. Os dados foram recolhidos através de entrevistas presenciais, utilizando o “Formulário de avaliação das caraterísticas descritivas das mulheres e da perceção das alterações climáticas” e a “Escala de ansiedade face às alterações climáticas para a saúde das mulheres”, entre junho e outubro de 2024, num centro de saúde familiar na província de Mersin, na Turquia. A análise dos dados incluiu métodos estatísticos descritivos e análise do detetor de interação automática do qui-quadrado.

**Resultados:**

A idade média das mulheres foi de 27,91 ± 8,30 anos (n = 351). A pontuação média da Escala de Ansiedade face às Alterações Climáticas para a Saúde das Mulheres foi de 52,46 ± 17,19. A experiência de ansiedade das mulheres em relação às consequências negativas das alterações climáticas afectou a escala e todas as subdimensões (saúde fisiológica, comportamento e género) (p < 0,05).

**Conclusão:**

Os profissionais de saúde devem fornecer informações e avaliações que incluam os efeitos das alterações climáticas na saúde nos serviços de prestação de cuidados. Devem planear intervenções para reduzir os níveis de ansiedade das mulheres em relação às alterações climáticas.

## INTRODUCTION

Climate change has been acknowledged as one of the biggest global health threats in the 21st century for our country and the world^(1)^. Although climate change is a common concern for humanity, the United Nations Framework Convention on Climate Change reports that its effects on women and men are frequently encountered and demonstrate differences^(2,3)^. A total of 1.3 billion people in low- and middle-income countries worldwide live below the poverty line and 70% of them are composed of women^(3)^. WHO and ACOG state that due to biological, political, and cultural factors, this situation causes women to be among the most vulnerable groups affected by climate change^(4)^. Data from Australia^(5)^, Uganda^(6)^, Southeast Asia^(7, 8, 9)^, and Canada^(10)^ indicate disproportional exposure of women to climate-related threats to their socio-economic position, livelihoods, food security, and health^(11)^. Therefore, unless appropriate adaptation measures are taken, this disparity is expected to worsen in the coming decades.

The impacts of climate change, such as food and water insecurity, extreme weather events, and spread of disease, could cause women in affected areas to face a high risk of disease, malnutrition, sexual violence, poor mental health, reproductive failure, adverse obstetric outcomes, and even death^(12)^. Due to their effects on reproductive and sexual health, toxic exposures may cause adverse obstetric outcomes such as infertility, abortus, preterm birth, and low birth weight newborns and neurodevelopmental delays such as autism and attention deficit hyperactivity disorder^(13,14)^. Besides the negative physical consequences, the psychological effects of climate change also lead to mental health problems such as anxiety, antisocial behaviors, and suicide attempts^(15, 16, 17)^.

For this reason, the impact of climate change on human health is important, especially in terms of its consequences for women’s health. Higher levels of women’s health in a society increase the possibility of growing up for healthy generations in that society and enhancing the welfare of society^(15,18)^. In this regard, it is necessary to know about the effects of climate change, one of the important problems in this age, especially on women’s health and to take measures. This study aims to determine women’s anxiety levels towards climate change and the affecting factors.

Research Questions

- What are the key factors affecting climate change anxiety in women of reproductive age?
- How do climate change anxiety levels in women of reproductive age differ based on independent variables (such as age, education level, marital status, knowing about climate change, following the news about climate change, and experiencing anxiety about the negative consequences of climate change, etc.)?

## METHOD

### Design of Study and Participants

This study is descriptive and cross-sectional. It was conducted in a family health center in Mersin province of Türkiye between June and October 2024. Mersin, located on the southern coast of Türkiye, holds strategic importance in the context of climate change due to its unique geographical, ecological, and socioeconomic characteristics. As part of the Mediterranean climate zone, the province is particularly vulnerable to the adverse impacts of climate change, including rising temperatures, decreasing precipitation, and increased frequency of extreme weather events. Given these factors, Mersin represents a critical area for climate change research.

A total of 4050 women at reproductive age were registered in the family health center between the specified dates. According to the sample calculation with a known population, the sample of the study consisted of 351 women (95% confidence interval and 0.05 margin of error). The study included women who were aged between 18 and 45, spoke Turkish, had no chronic and psychological disorders, and agreed to participate in the study. Women who were diagnosed with any health problems were excluded from the study.

### Data Collection

Data were collected through the ‘Personal Information Form’ and the ‘Climate Change Anxiety Scale for Women’s Health’.

### Personal Information Form

The Personal Information Form was prepared by the researcher and consisted of 23 questions including women’s descriptive characteristics and climate-related knowledge^(7,13,16,19)^.

### Climate Change Anxiety Scale for Women’s Health

The scale was developed by Süğüt and Vurgeç^(19)^. The Climate Change Anxiety Scale for Women’s Health consists of 18 items responded on a 5-point Likert scale. The scale consists of 3 sub-scales, which include physiological health, behavior, and gender. The score obtained from each sub-scale indicates anxiety related to that sub-scale. The scores to be obtained from the scale range between 18 and 90, with higher total scores indicating women’s increased anxiety about climate change. Cronbach’s alpha value of the original scale was found 0.96, and Cronbach’s alpha value was found 0.93 in this study.

### Data Analysis and Treatment

Data were analyzed using IBM SPSS Version 23. Data analysis included using numbers, percentages, means, min-max, and standard deviation values as descriptive statistical methods. CHAID analysis, one of the data mining methods, was performed to categorize the independent variables. Of the existing data mining methods, CHAID analysis is the most preferred decision tree methodology for classification and regression models. It uses large samples to put forward potentially highly reliable estimates and can predict missing observations in independent variables^(20)^. CHAID analysis reveals the effects of missing data in the model more effectively than other decision tree methods^(21)^. The tree diagram for a CHAID analysis is drawn by selecting the most important relationships, thus dividing the dependent variable into detailed homogeneous subnodes to increase prediction accuracy^(20)^. One of the important features of CHAID analysis is that it detects the common effect between independent variables^(22)^. All independent variables were included in the model. The independent variables that yielded results in the current analysis included knowing about climate change, following the news about climate change, and experiencing anxiety about the negative consequences of climate change. The statistical significance level was accepted as *p* < 0.05.

### Ethical Aspects

Before the study was conducted, approval was obtained from the non-interventional clinical research ethics committee of Tarsus University (dated 28/03/2024 and number: 2024/30), and permission was obtained from Mersin Provincial Health Directorate (dated 13/06/2024 and number: 61). Informed consent was obtained from the participating women, and they were told that they could leave the study at any time.

## RESULTS

### Descriptive Characteristics of the Participants

Of all the participating women, 33.6% were married, 66.4% were single, 52.7% were employed, 78.3% had a university education and above, and 63.0% reportedly had income equal to expenses. Besides, 90.9% of the partners had a university education and above, and the majority of the partners were employed. It was also found that 80.1% of the women had regular menstruation and most of them were not in menopause. Besides, 66.1% of the women did not have allergies, 67.5% were informed about climate change, 68.1% followed the news about climate change, and 79.2% mentioned the negative effects of climate change on women’s health. While 59.0% of women were anxious about the negative consequences of climate change, 69.2% thought that they were affected by climate change, and 71.2% reported an increase in the frequency of getting sick due to climate change. The average age of the women was 27.91 ± 8.30, the average age of menarche was 13.05 ± 1.43, the average menstrual cycle interval was 28.06 ± 8.14, and the average number of pregnancies, live births, stillbirths and abortions were 0.68 ± 1.17, 0.54 ± 0.92, 0.04 ± 0.27, and 0.12 ± 0.46, respectively (Table 1).

**Table 1:** Distribution of women according to their descriptive characteristics and perceptions of climate change – Mersin, Türkiye, 2024.

	(n = 351)	(%)
Marital status		
Married	118	33.6
Single	233	66.4
Employment status		
Employed	185	52.7
Unemployed	166	47.3
Education level		
Secondary school	76	21.7
University and above	275	78.3
Income level		
Income less than expenses	98	27.9
Income equal to expenses	221	63.0
Income more than expenses	32	9.1
Spouse’s education level		
Secondary school	32	9.1
University and above	319	90.9
Spouse’s employment status		
Employed	342	97.4
Unemployed	9	2.6
Regular Menstruation		
Yes	281	80.1
No	70	19.9
Menopause status		
Yes	15	4.3
No	336	95.7
Presence of allergies		
Yes	119	33.9
No	232	66.1
Knowing about climate change		
Yes	237	67.5
No	114	32.5
Following the news about climate change		
Yes	239	68.1
No	112	31.9
Effect of climate change on women’s health		
Yes	278	79.2
No	73	20.8
Experiencing anxiety about the negative consequences of climate change		
Yes	207	59.0
No	144	41.0
Thinking about being affected by climate change		
Yes	243	69.2
No	108	30.8
Does climate change increase the frequency of getting sick?		
Yes	250	71.2
No	101	28.8
	**Mean ± SD**	
Age	27.91 ± 8.30	
Age of menarche	13.05 ± 1.43	
Menstruation duration (days)	28.06 ± 8.14	
Number of pregnancies	0.68 ± 1.17	
Number of living children	0.54 ± 0.92	
Number of stillbirths	0.04 ± 0.27	
Number of abortions	0.12 ± 0.46	

### Decision Tree for the Climate Change Anxiety Scale for Women’s Health

The decision tree analysis based on the Climate Change Anxiety Scale for Women’s Health total scores showed that the average anxiety score at the first node (Node 0) was 52.459 ± 17.194. In the first split based on the state of experiencing anxiety about the negative consequences of climate change, the anxiety mean score of those who answered ‘Yes’ was 60.758 ± 15.228, and the anxiety mean score of those who answered ‘No’ was 40.528 ± 12.103. Experiencing anxiety about the negative consequences of climate change was found to have a significant effect on the Climate Change Anxiety Scale for Women’s Health total score (F = 176.529; *P* < 0.001).

**Figure 1:**
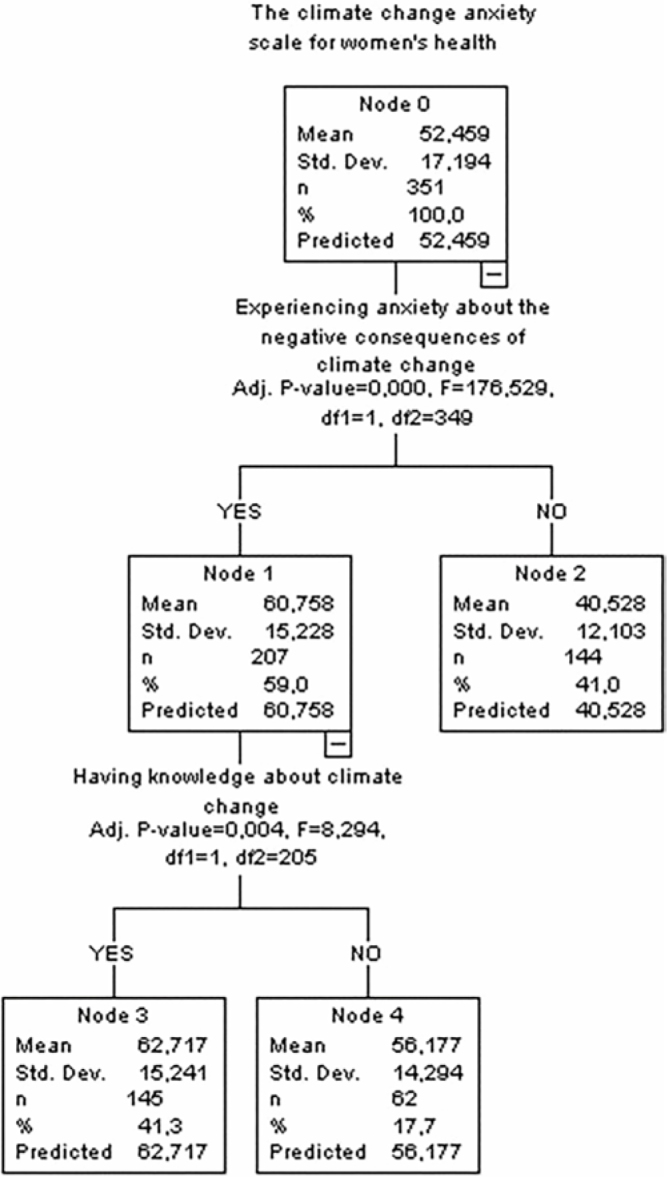
Decision tree for the climate change anxiety scale for women’s health total score.

A second split was performed according to whether the participants knew about climate change or not for the individuals who answered ‘Yes’ in the case of experiencing anxiety. The anxiety mean score of those who knew about climate change was 62.717 ± 15.241, while the anxiety mean score of those who did not know about it was 56.177 ± 14.294. Knowing about climate change was found to affect the total score (F = 8.294; *P* = 0.004). These results show that individuals who experience anxiety about the negative consequences of climate change and know about climate change have higher levels of anxiety (Figure 1).

### Decision Tree for the Physiological Health Score

This decision tree diagram analyzed the distribution of physiological health scores according to various variables. Starting from Node 0, the average physiological health score of the women in the sample was calculated as 22.821 ± 9.657. The first split was based on the individuals’ anxiety towards the negative consequences of climate change. A difference was detected in the physiological health score according to the individuals’ experience of anxiety towards the negative consequences of climate change (F = 276.671; *P* < 0.001). While the average physiological health mean score was 28.169 ± 7.346 for those who answered ‘Yes’ (Node 1), it was 15.132 ± 7.042 for those who answered ‘No’ (Node 2).

**Figure 2:**
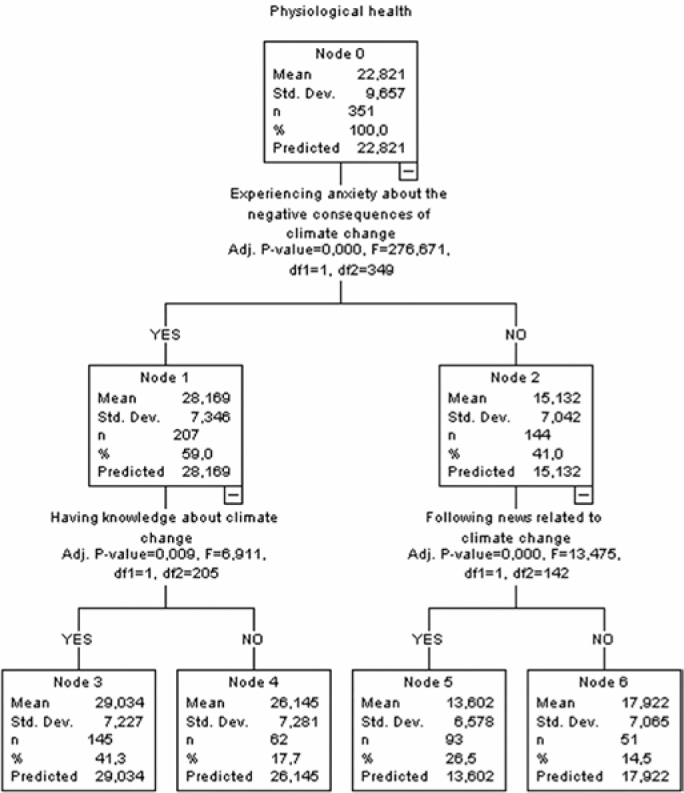
Decision tree for the physiological health score.

A second split was performed based on Node 1, which evaluated whether the individuals knew about climate change or not. The physiological health mean score of those who answered ‘Yes’ (Node 3) was 29.034 ± 7.227, while that of those who answered ‘No’ (Node 4) was 26.145 ± 7.281. Knowing about climate change was found to affect the score (F = 6.911; *P* = 0.009). A third split was performed based on Node 2, which analyzed whether the individuals followed the news about climate change or not. The physiological health mean score was found to be 13.602 ± 6.578 in individuals who followed the news (Node 5) and 17.922 ± 7.065 in those who did not follow the news (Node 6). The mean scores were found to differ according to following the news (F = 13.475; *P* < 0.001). These results show that anxiety about the negative consequences of climate change, knowing about it, and following the news on the issue have a significant effect on physiological health. Physiological health scores were found to be higher, especially in individuals who had a high anxiety level and knew about the issue (Figure 2).

### Decision Tree for Behavior Score

In this decision tree diagram, individuals’ behavior scores were analyzed according to various variables. In Node 0, women’s behavior mean score was calculated as 16.732 ± 5.819. The first split was performed based on individuals’ anxiety about the negative consequences of climate change. While the behavior mean score (Node 1) was 18.155 ± 5.566 in individuals who experienced anxiety, it was 14.688 ± 5.577 in individuals who did not (Node 2). Women’s anxiety about the negative consequences of climate change was found to have a statistically significant effect on behavior (F = 32.897; *P* < 0.001).

**Figure 3:**
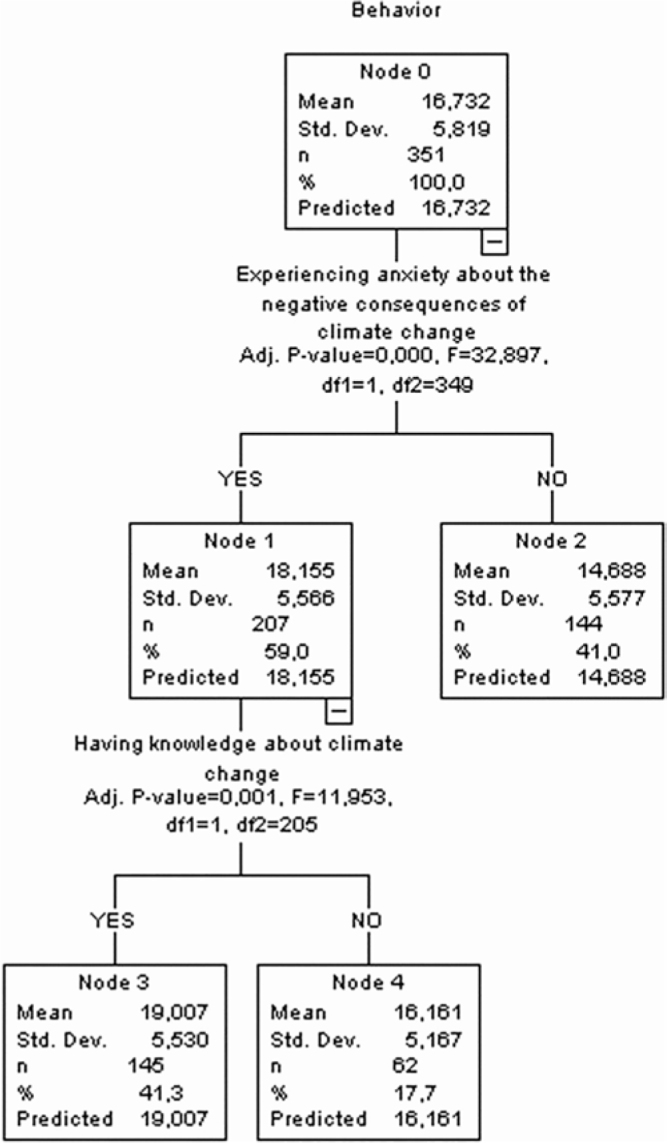
Decision tree for behavior score.

A second split was performed based on Node 1, which analyzed whether the individuals knew about climate change or not. While the behavior mean score of individuals who knew about climate change (Node 3) was 19.007 ± 5.530, the mean score of individuals who did not know about it (Node 4) was 16.161 ± 5.167. Behavior scores were found to differ according to knowing about climate change (F = 11.953; *P* = 0.001). The results showed that anxiety and knowledge about climate change had a significant effect on individuals’ behavior scores. Behavior scores were found to be higher, particularly in individuals who experienced anxiety about climate change and knew about the issue (Figure 3).

### Decision Tree for Gender Score

**Figure 4:**
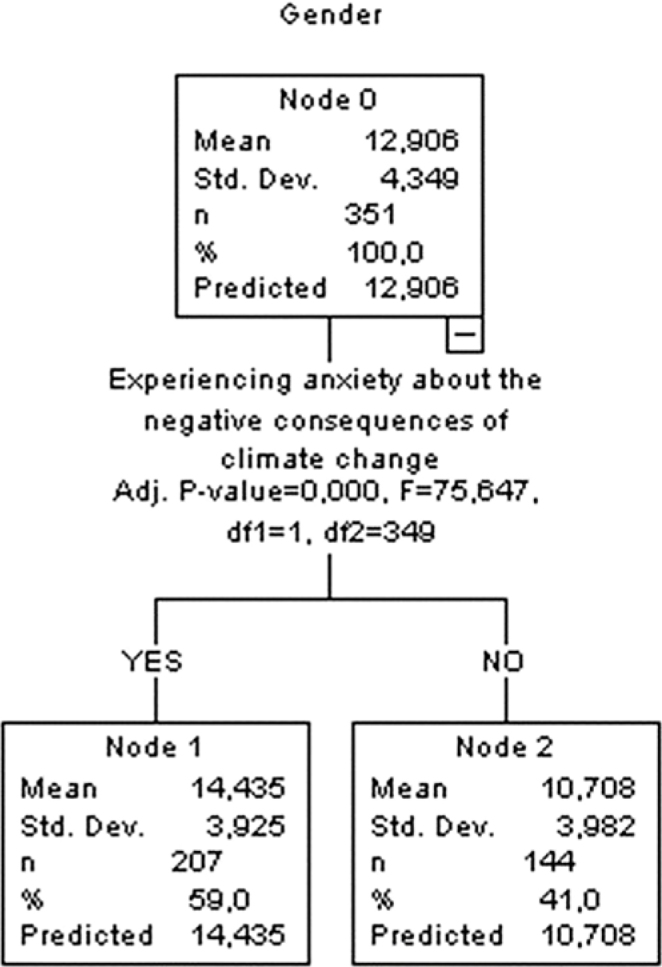
Decision tree for gender score.

Analysis results showed that the women’s gender mean score was found to be 12.906 ± 4.349 in Node 0. In Node 1, where individuals who expressed anxiety about the negative consequences of climate change (saying yes) were found, the anxiety mean score was found to be 14.435 ± 3.925. On the other hand, in Node 2, where individuals who did not experience anxiety about climate change (who said no) were found, the anxiety mean score was found to be 10.708 ± 3.982. The results of the analysis showed that the anxiety mean score of individuals who experienced anxiety about the negative consequences of climate change was significantly higher compared to individuals who did not experience anxiety (F = 75.647, *P* < 0.001) (Figure 4).

## DISCUSSION

The climate change crisis, one of the most important global problems of our time, has direct or indirect effects on women’s health. Increasing climate change also causes some anxiety and worries about the health of both women and future generations. The purpose of this study is to determine women’s anxiety levels about climate change and the affecting factors.

The participants’ Climate Change Anxiety Scale for Women’s Health mean score was found 52.46 ± 17.19 in this study, indicating a moderate level of anxiety. Hamlaçi Başkaya et al.^(23)^ reported the Climate Change Anxiety Scale for Women’s Health as 55.89 ± 17.12. Demir et al.^(24)^ detected a significant relationship between women’s awareness of climate change and their anxiety about this issue. Current and future uncertainties about the effects of climate change on women and their environment may have increased women’s anxiety levels.

The results of CHAID analysis in this study showed that the variable affecting the level of anxiety in the scale and all its sub-scales was the state of experiencing anxiety about the negative consequences of climate change (Figures 1–4). Korkmaz and Sahin reported that 60% of the individuals thought that climate change affected life negatively and 49.3% were concerned about climate change^(25)^. Demir et al.^(24)^ highlighted that women’s concern about environmental threats correlates with their anxiety regarding personal and societal well-being. The literature emphasizes the effects of climate change on women’s health, indicating that individuals who have high awareness of climate change and environmental events have higher levels of anxiety, worry, and stress and increased awareness may lead to anxiety in women and may affect their psychological well-being and mental health both directly and indirectly^(15,26)^. All these research results suggest that women’s anxiety levels increase due to their high level of awareness about the negative consequences of climate change.

This study found that following the news about climate change had a significant effect on the physiological health sub-scale (Figure 2). In particular, the decision tree for physiological health (Figure 2) indicates that both awareness of climate change and media consumption significantly affect this sub-scale. Demir et al.^(24)^ reported that most of the women in their study followed the news about climate change and obtained most of the information from sources such as television and social media/Internet. Studies show that people mostly access climate change news through digital media such as television and social media/Internet^(15,27)^. Maran and Begoti^(28)^ reported that women follow information on climate change several times a week and that their sources of information were mostly Facebook, printed and online newspapers, TV news and programs, and family and friend circles. The results of this study showed that people knew about climate change and followed the news from different platforms, which may affect their level of anxiety about climate change.

CHAID analysis in this study showed that knowing about climate change was one of the variables affecting the Climate Change Anxiety Scale for Women’s Health and physiological health and behavior sub-scales (Figures 1–3). Within the scope of the perceptions and opinions of the participants in Korkmaz and Şahin’s study, significant differences (*P* < 0.05) were detected in terms of awareness of the effects of climate change on their lives and the level of knowledge about climate change^(25)^. The behavioral dimension (Figure 3) also reflects that individuals informed about climate change are more likely to engage in protective behaviors, echoing results from Stone et al.^(29)^, who linked awareness with proactive mental and physical health strategies in women. Süğüt and Vurgeç^(19)^ reported that when women were asked whether they knew anything about ‘climate change’, 39.8% reportedly knew about it and 39.7% partially knew about it. Knowing about climate change actually depends on how women perceive the information and how they cope with this information. These reasons may vary in women’s anxiety levels.

Figure 4, which focuses on the gender-specific dimension of climate change anxiety, underscores the heightened psychological burden experienced by women. This finding is in line with reports by the WHO and ACOG, which highlight the intersectionality between gender and environmental stressors, particularly among vulnerable populations^(4,5)^. Taken together, these results suggest that women’s anxiety levels related to climate change are shaped by a complex interplay of factors, including awareness, access to information, and individual perceptions, as supported by an expanding body of international literature.

Although global scholarly interest in the intersection of climate change and mental health has increased, empirical research focusing specifically on climate change-related anxiety among women of reproductive age remains scarce, particularly in the context of low and middle-income countries such as Türkiye. Moreover, culturally embedded gender roles and societal expectations regarding environmental responsibility may shape women’s cognitive and emotional responses to climate-related threats in unique ways. To elucidate the underlying mechanisms and establish causal relationships, future studies should consider employing longitudinal designs and incorporating objective biomarkers of stress and health outcomes.

### Strengths and Limitations of Study

This study possesses several noteworthy strengths. Primarily, it contributes to a relatively underexplored area by focusing on climate change anxiety among women of reproductive age in Türkiye, thereby addressing a significant gap in the existing body of literature. Furthermore, the utilization of CHAID analysis, a sophisticated and widely accepted decision tree method, enabled an in-depth examination of the multifaceted relationships among variables and facilitated the identification of complex interaction patterns that influence anxiety levels.

Nevertheless, the study is not without limitations. The cross sectional design restricts the ability to infer causal relationships between variables. Additionally, data collection was confined to a single family health center located in Mersin, which may limit the external validity and generalizability of the findings to broader populations.

## CONCLUSION

Knowing about climate change, following the news about climate change, and experiencing anxiety about the negative consequences of climate change were found to be the main factors affecting the level of anxiety about climate change in women at reproductive age. Knowing about the effects of climate change, which is one of the important problems of today, and determining the affecting underlying factors are highly important in order to eliminate the concerns on this issue and to take measures. Investing in women’s health is investing in the health of current and future generations. The development of policies and actions at national and international levels should be supported to minimize concerns about climate change. During their care services, health professionals should provide information on the effects of climate change on health.

## Data Availability

The datasets used and analyzed during the study are available from the corresponding author on reasonable request.
