# Supplementary material for: Factors relating to climate change anxiety in women at reproductive age: A CHAID analysis
Source: Rev Esc Enferm USP. 2025 Sep 8;59:e20250182. doi: 10.1590/1980-220X-REEUSP-2025-0182en (PMC12448239; doi:10.1590/1980-220X-REEUSP-2025-0182en)
Supplement: Translation

# Translation: Factores relacionados con la ansiedad ante el cambio climático en mujeres en edad reproductiva: Un análisis CHAID

**DOI:** 10.1590/1980-220X-REEUSP-2025-0182es

**Authors:** Tuğçe Sönmez, Enes Karaman

**Affiliations:** 1 Tarsus University, Faculty of Health Sciences, Department of Midwifery, Mersin, Türkiye.; 2 Niğde Ömer Halisdemir University, Faculty of Medicine, Department of Obstetrics and Gynaecology, Niğde, Türkiye.

**Keywords:** Ansiedad, Cambio climático, Partería, Reproducción, Salud de la mujer

## Abstract

**Objetivo:**

Este estudio pretendía determinar los factores relacionados con la ansiedad ante el cambio climático en mujeres en edad reproductiva.

**Método:**

Este estudio es descriptivo y transversal. En él participaron 351 mujeres de entre 18 y 45 años. Los datos se recopilaron mediante entrevistas cara a cara utilizando el «Formulario de evaluación de las características descriptivas y la percepción del cambio climático de las mujeres» y la «Escala de ansiedad ante el cambio climático para la salud de las mujeres» entre junio y octubre de 2024 en un centro de salud familiar de la provincia de Mersin (Turquía). El análisis de datos incluyó métodos estadísticos descriptivos y análisis de detector automático de ınteracción chi-cuadrado.

**Resultados:**

La edad media de las mujeres era de 27,91 ± 8,30 años (n = 351). La puntuación media de la Escala de Ansiedad ante el Cambio Climático para la Salud de la Mujer fue de 52,46 ± 17,19. La experiencia de ansiedad de las mujeres respecto a las consecuencias negativas del cambio climático afectó a la escala y a todas las subdimensiones (salud fisiológica, comportamiento y género) (p < 0,05).

**Conclusiones:**

Los profesionales sanitarios deben proporcionar información y evaluaciones que incluyan los efectos del cambio climático sobre la salud en los servicios asistenciales. Deberían planificar intervenciones para reducir los niveles de ansiedad de las mujeres ante el cambio climático.

## INTRODUCCIÓN

El cambio climático ha sido reconocido como una de las mayores amenazas para la salud mundial en el siglo XXI, tanto para nuestro país como para el resto del mundo^(1)^. Aunque el cambio climático es una preocupación común para toda la humanidad, la Convención Marco de las Naciones Unidas sobre el Cambio Climático señala que sus efectos sobre las mujeres y los hombres son frecuentes y muestran diferencias^(2,3)^. Un total de 1300 millones de personas en países de ingresos bajos y medios de todo el mundo viven por debajo del umbral de la pobreza y el 70% de ellas son mujeres^(3)^. La OMS y el Colegio Americano de Obstetricia y Ginecología (ACOG) afirman que, debido a factores biológicos, políticos y culturales, esta situación hace que las mujeres se encuentren entre los grupos más vulnerables afectados por el cambio climático^(4)^. Los datos de Australia^(5)^, Uganda^(6)^, el sudeste asiático^(7, 8, 9)^ y Canadá^(10)^ indican una exposición desproporcionada de las mujeres a las amenazas relacionadas con el clima para su posición socioeconómica, sus medios de vida, la seguridad alimentaria y la salud^(11)^. Por lo tanto, a menos que se adopten medidas de adaptación adecuadas, se prevé que esta disparidad empeore en las próximas décadas.

Los efectos del cambio climático, como la inseguridad alimentaria y del agua, los fenómenos meteorológicos extremos y la propagación de enfermedades, podrían hacer que las mujeres de las zonas afectadas se enfrentaran a un alto riesgo de enfermedad, malnutrición, violencia sexual, mala salud mental, problemas reproductivos, resultados obstétricos adversos e incluso la muerte^(12)^. Debido a sus efectos sobre la salud reproductiva y sexual, la exposición a sustancias tóxicas puede provocar resultados obstétricos adversos, como infertilidad, abortos, partos prematuros y recién nacidos con bajo peso al nacer, así como retrasos en el desarrollo neurológico, como el autismo y el trastorno por déficit de atención con hiperactividad^(13,14)^. Además de las consecuencias físicas negativas, los efectos psicológicos del cambio climático también provocan problemas de salud mental, como ansiedad, comportamientos antisociales e intentos de suicidio^(15, 16, 17)^.

Por esta razón, el impacto del cambio climático en la salud humana es importante, especialmente en lo que respecta a sus consecuencias para la salud de las mujeres. Un mayor nivel de salud de las mujeres en una sociedad aumenta la posibilidad de que crezcan generaciones sanas en esa sociedad y de mejorar el bienestar de la misma^(15,18)^. En este sentido, es necesario conocer los efectos del cambio climático, uno de los problemas importantes de nuestra época, especialmente en la salud de las mujeres, y tomar medidas al respecto. El objetivo de este estudio es determinar los niveles de ansiedad de las mujeres ante el cambio climático y los factores que lo influyen.

Preguntas de investigación

- ¿Cuáles son los factores clave que influyen en la ansiedad por el cambio climático en las mujeres en edad reproductiva?
- ¿Cómo varían los niveles de ansiedad por el cambio climático en las mujeres en edad reproductiva en función de variables independientes (como la edad, el nivel de educación, el estado civil, el conocimiento sobre el cambio climático, el seguimiento de las noticias sobre el cambio climático y la experiencia de ansiedad por las consecuencias negativas del cambio climático, etc.)?

## MÉTODO

### Diseño del Estudio y Participantes

Este estudio es descriptivo y transversal. Se llevó a cabo en un centro de salud familiar de la provincia de Mersin, en Turquía, entre junio y octubre de 2024. Mersin, situada en la costa sur de Turquía, tiene una importancia estratégica en el contexto del cambio climático debido a sus características geográficas, ecológicas y socioeconómicas únicas. Como parte de la zona climática mediterránea, la provincia es especialmente vulnerable a los efectos adversos del cambio climático, como el aumento de las temperaturas, la disminución de las precipitaciones y el aumento de la frecuencia de fenómenos meteorológicos extremos. Teniendo en cuenta estos factores, Mersin representa una zona crítica para la investigación sobre el cambio climático.

Un total de 4050 mujeres en edad reproductiva se registraron en el centro de salud familiar entre las fechas especificadas. Según el cálculo de la muestra con una población conocida, la muestra del estudio estaba formada por 351 mujeres (intervalo de confianza del 95% y margen de error del 0,05). El estudio incluyó a mujeres de entre 18 y 45 años, que hablaban turco, no padecían trastornos crónicos ni psicológicos y aceptaron participar en el estudio. Se excluyó del estudio a las mujeres a las que se les diagnosticó algún problema de salud.

### Recolección de Datos

Los datos se recolectaron a través del «Formulario de información personal» y la «Escala de ansiedad por el cambio climático para la salud de la mujer».

### Formulario de Información Personal

El formulario de información personal fue elaborado por el investigador y constaba de 23 preguntas que incluían características descriptivas de las mujeres y conocimientos relacionados con el clima^(7,13,16,19)^.

### Escala de Ansiedad por el Cambio Climático Para la Salud de la Mujer (Climate Change Anxiety Scale for Women’s Health)

La escala fue desarrollada por Süğüt y Vurgeç^(19)^. La Escala de Ansiedad por el Cambio Climático para la Salud de la Mujer consta de 18 ítems que se responden en una escala Likert de 5 puntos. La escala consta de 3 subescalas, que incluyen la salud fisiológica, el comportamiento y el género. La puntuación obtenida en cada subescala indica la ansiedad relacionada con dicha subescala. Las puntuaciones que se obtienen en la escala oscilan entre 18 y 90, y las puntuaciones totales más altas indican un aumento de la ansiedad de las mujeres respecto al cambio climático. El valor alfa de Cronbach de la escala original fue de 0,96, y el valor alfa de Cronbach en este estudio fue de 0,93.

### Análisis y Tratamiento de los Datos

Los datos se analizaron utilizando IBM SPSS Versión 23. El análisis de los datos incluyó el uso de números, porcentajes, medias, valores mínimos y máximos y desviación estándar como métodos estadísticos descriptivos. Se realizó un análisis *Chi-square automatic interaction detection - CHAID* (Detección automática de interacción mediante la prueba de chi cuadrado), uno de los métodos de minería de datos, para categorizar las variables independientes. De los métodos de minería de datos existentes, el análisis CHAID es la metodología de árbol de decisión más preferida para los modelos de clasificación y regresión. Utiliza muestras grandes para presentar estimaciones potencialmente muy fiables y puede predecir observaciones faltantes en variables independientes^(20)^. El análisis CHAID revela los efectos de los datos faltantes en el modelo de manera más eficaz que otros métodos de árboles de decisión^(21)^. El diagrama de árbol para un análisis CHAID se dibuja seleccionando las relaciones más importantes, dividiendo así la variable dependiente en subnodos homo-géneos detallados para aumentar la precisión de la predicción^(20)^. Una de las características importantes del análisis CHAID es que detecta el efecto común entre variables independientes^(22)^. Se incluyeron todas las variables independientes en el modelo. Las variables independientes que dieron resultados en el análisis actual incluyeron el conocimiento sobre el cambio climático, el seguimiento de las noticias sobre el cambio climático y la experiencia de ansiedad por las consecuencias negativas del cambio climático. Se aceptó como nivel de significación estadística *p* < 0,05.

### Aspectos Éticos

Antes de realizar el estudio, se obtuvo la aprobación del comité de ética de investigación clínica no intervencionista de la Universidad de Tarsus (con fecha del 28/03/2024 y número: 2024/30), y se obtuvo el permiso de la Dirección Provincial de Salud de Mersin (con fecha del 13/06/2024 y número: 61). Se obtuvo el consentimiento informado de las mujeres participantes y se les informó de que podían abandonar el estudio en cualquier momento.

## RESULTADOS

### Características Descriptivas de las Participantes

De todas las mujeres participantes, el 33,6% estaban casadas, el 66,4% eran solteras, el 52,7% tenían empleo, el 78,3% tenían estudios universitarios o superiores y el 63,0% declararon tener ingresos iguales a los gastos. Además, el 90,9% de las parejas tenían estudios universitarios o superiores y la mayoría de las parejas tenían empleo. También se observó que el 80,1% de las mujeres tenían menstruación regular y la mayoría de ellas no estaban en la menopausia. Además, el 66,1% de las mujeres no tenían alergias, el 67,5% estaban informadas sobre el cambio climático, el 68,1% seguían las noticias sobre el cambio climático y el 79,2% mencionaron los efectos negativos del cambio climático en la salud de las mujeres. Mientras que el 59,0% de las mujeres estaban preocupadas por las consecuencias negativas del cambio climático, el 69,2% pensaban que se veían afectadas por el cambio climático y el 71,2% informaron de un aumento en la frecuencia de enfermedades debido al cambio climático. La edad media de las mujeres era de 27,91 ± 8,30, la edad media de la menarquia era de 13,05 ± 1,43, el intervalo medio del ciclo menstrual era de 28,06 ± 8,14, y el número medio de embarazos, nacidos vivos, mortinatos y abortos era de 0,68 ± 1,17, 0,54 ± 0,92, 0,04 ± 0,27 y 0,12 ± 0,46, respectivamente (Tabla 1).

**Tabla 1:** Distribución de las mujeres según sus características descriptivas y percepciones sobre el cambio climático - Mersin, Turquía, 2024.

	(n = 351)	(%)
Estado civil		
Casada	118	33.6
Soltera	233	66.4
Ocupación		
Empleada	185	52.7
Desempleada	166	47.3
Nivel educativo		
Secundario	76	21.7
Universidad y superior	275	78.3
Nivel de ingresos		
Ingresos menos que gastos	98	27.9
Ingresos igual a gasto	221	63.0
Ingresos mayores que gastos	32	9.1
Nivel educativo del compañero		
Seccundario	32	9.1
Universidad y superior	319	90.9
Ocupación del compañero		
Empleado	342	97.4
Desempleado	9	2.6
Menstrución regular		
Si	281	80.1
No	70	19.9
Menopausia		
Si	15	4.3
No	336	95.7
Presencia de alergias		
Si	119	33.9
No	232	66.1
Conocimiento de cambio climático		
Si	237	67.5
No	114	32.5
Sigue las noticcias de cambio climático		
Si	239	68.1
No	112	31.9
Efectos del cambio climático en la salud de la mujer		
Si	278	79.2
No	73	20.8
Experimenta ansiedad sobre las consecuencias negativas del cambio climáatico		
Si	207	59.0
No	144	41.0
Piensa sobre ser afectada por el cambio climático		
Si	243	69.2
No	108	30.8
¿El cambio climático aumenta sus chances de enfermar?		
Si	250	71.2
No	101	28.8
	**Media ± DE**	
Edad	27.91 ± 8.30	
Edad de menarca	13.05 ± 1.43	
Duración de la menstruación(días)	28.06 ± 8.14	
Número de embarazos	0.68 ± 1.17	
Número de hijos vivos	0.54 ± 0.92	
Número de mortinatos	0.04 ± 0.27	
Número de abortos	0.12 ± 0.46	

### Árbol de Decisión Para la Escala de Ansiedad por el Cambio Climático en la Salud de las Mujeres

El análisis del árbol de decisión basado en las puntuaciones totales de la Escala de Ansiedad por el Cambio Climático en la Salud de las Mujeres mostró que la puntuación media de ansiedad en el primer nodo (Nodo 0) era de 52,459 ± 17,194. En la primera división basada en el estado de ansiedad ante las consecuencias negativas del cambio climático, la puntuación media de ansiedad de quienes respondieron «Sí» fue de 60,758 ± 15,228, y la puntuación media de ansiedad de quienes respondieron «No» fue de 40,528 ± 12,103. Se observó que experimentar ansiedad por las consecuencias negativas del cambio climático tenía un efecto significativo en la puntuación total de la Escala de Ansiedad por el Cambio Climático para la Salud de la Mujer (F = 176,529; *P* < 0,001).

Se realizó una segunda división según si las participantes conocían o no el cambio climático en el caso de las personas que respondieron «Sí» a la pregunta sobre si experimentaban ansiedad. La puntuación media de ansiedad de las que conocían el cambio climático fue de 62,717 ± 15,241, mientras que la puntuación media de ansiedad de las que no lo conocían fue de 56,177 ± 14,294. Se observó que el conocimiento del cambio climático afectaba a la puntuación total (F = 8,294; *P* = 0,004). Estos resultados muestran que las personas que experimentan ansiedad por las consecuencias negativas del cambio climático y conocen el cambio climático tienen niveles más altos de ansiedad (Figura 1).

**Figura 1:** Árbol de decisión para la escala de ansiedad por el cambio climático para la puntuación total de salud de las mujeres.

### Árbol de Decisión Para la Puntuación de Salud Fisiológica

Este diagrama de árbol de decisión analizó la distribución de las puntuaciones de salud fisiológica según diversas variables. Partiendo del nodo 0, se calculó que la puntuación media de salud fisiológica de las mujeres de la muestra era de 22,821 ± 9,657. La primera división se basó en la ansiedad de las personas hacia las consecuencias negativas del cambio climático. Se detectó una diferencia en la puntuación de salud fisiológica según la experiencia de ansiedad de las personas hacia las consecuencias negativas del cambio climático (F = 276,671; *P* < 0,001). Mientras que la puntuación media de salud fisiológica fue de 28,169 ± 7,346 para quienes respondieron «Sí» (Nodo 1), fue de 15,132 ± 7,042 para quienes respondieron «No» (Nodo 2).

Se realizó una segunda división basada en el Nodo 1, que evaluaba si los individuos conocían o no el cambio climático. La puntuación media de salud fisiológica de quienes respondieron «Sí» (Nodo 3) fue de 29,034 ± 7,227, mientras que la de quienes respondieron «No» (Nodo 4) fue de 26,145 ± 7,281. Se observó que el conocimiento sobre el cambio climático influía en la puntuación (F = 6,911; *P* = 0,009). Se realizó una tercera división basada en el nodo 2, que analizaba si los individuos seguían las noticias sobre el cambio climático o no. Se observó que la puntuación media de salud fisiológica era de 13,602 ± 6,578 en los individuos que seguían las noticias (nodo 5) y de 17,922 ± 7,065 en los que no las seguían (nodo 6). Se observó que las puntuaciones medias diferían en función de si se seguían las noticias (F = 13,475; *P* < 0,001). Estos resultados muestran que la ansiedad por las consecuencias negativas del cambio climático, el conocimiento del mismo y el seguimiento de las noticias sobre el tema tienen un efecto significativo en la salud fisiológica. Se observó que las puntuaciones de salud fisiológica eran más altas, especialmente en las personas que tenían un nivel de ansiedad elevado y conocían el tema (Figura 2).

**Figura 2:** Árbol de decisión para la puntuación de salud fisiológica.

### Árbol de Decisión Para la Puntuación de Comportamiento

En este diagrama de árbol de decisión, se analizaron las puntuaciones de comportamiento de los individuos según diversas variables. En el nodo 0, la puntuación media de comportamiento de las mujeres se calculó en 16,732 ± 5,819. La primera división se realizó en función de la ansiedad de los individuos ante las consecuencias negativas del cambio climático. Mientras que la puntuación media de comportamiento (nodo 1) fue de 18,155 ± 5,566 en los individuos que experimentaban ansiedad, fue de 14,688 ± 5,577 en los que no la experimentaban (nodo 2). Se observó que la ansiedad de las mujeres sobre las consecuencias negativas del cambio climático tenía un efecto estadísticamente significativo sobre el comportamiento (F = 32,897; *P* < 0,001).

Se realizó una segunda división basada en el Nodo 1, que analizó si los individuos conocían o no el cambio climático. Mientras que la puntuación media del comportamiento de los individuos que conocían el cambio climático (Nodo 3) fue de 19,007 ± 5,530, la puntuación media de los individuos que no lo conocían (Nodo 4) fue de 16,161 ± 5,167. Se observó que las puntuaciones de comportamiento diferían en función del conocimiento sobre el cambio climático (F = 11,953; *P* = 0,001). Los resultados mostraron que la ansiedad y el conocimiento sobre el cambio climático tenían un efecto significativo en las puntuaciones de comportamiento de los individuos. Se observó que las puntuaciones de comportamiento eran más altas, especialmente en los individuos que experimentaban ansiedad por el cambio climático y conocían el tema (Figura 3).

**Figura 3:** Árbol de decisión para la puntuación del comportamiento.

### Árbol de Decisión Para la Puntuación de Género

Los resultados del análisis mostraron que la puntuación media de género de las mujeres era de 12,906 ± 4,349 en el nodo 0. En el nodo 1, donde se encontraron personas que expresaron ansiedad por las consecuencias negativas del cambio climático (respondiendo «sí»), la puntuación media de ansiedad fue de 14,435 ± 3,925. Por otro lado, en el nodo 2, donde se encontraron personas que no experimentaban ansiedad por el cambio climático (que respondieron «no»), la puntuación media de ansiedad fue de 10,708 ± 3,982. Los resultados del análisis mostraron que la puntuación media de ansiedad de las personas que experimentaban ansiedad por las consecuencias negativas del cambio climático era significativamente mayor en comparación con las personas que no experimentaban ansiedad (F = 75,647, *P* < 0,001) (Figura 4).

**Figura 4:** Árbol de decisión para la puntuación de género.

## DISCUSIÓN

La crisis del cambio climático, uno de los problemas mundiales más importantes de nuestro tiempo, tiene efectos directos o indirectos sobre la salud de las mujeres. El aumento del cambio climático también provoca cierta ansiedad y preocupación por la salud tanto de las mujeres como de las generaciones futuras. El objetivo de este estudio es determinar los niveles de ansiedad de las mujeres respecto al cambio climático y los factores que lo influyen.

La puntuación media de la Escala de Ansiedad por el Cambio Climático para la Salud de la Mujer de las participantes en este estudio fue de 52,46 ± 17,19, lo que indica un nivel moderado de ansiedad. Hamlaçi Başkaya et al.^(23)^ informaron de una puntuación de 55,89 ± 17,12 en la Escala de Ansiedad por el Cambio Climático para la Salud de la Mujer. Demir et al.^(24)^ detectaron una relación significativa entre la conciencia de las mujeres sobre el cambio climático y su ansiedad sobre este tema. Las incertidumbres actuales y futuras sobre los efectos del cambio climático en las mujeres y su entorno pueden haber aumentado los niveles de ansiedad de las mujeres.

Los resultados del análisis CHAID en este estudio mostraron que la variable que afectaba al nivel de ansiedad en la escala y en todas sus subescalas era el estado de ansiedad ante las consecuencias negativas del cambio climático (Figura 1–4). Korkmaz y Sahin informaron de que el 60% de las personas pensaban que el cambio climático afectaba negativamente a la vida y el 49,3% estaban preocupadas por el cambio climático^(25)^. Demir et al.^(24)^ destacaron que la preocupación de las mujeres por las amenazas medioambientales se correlaciona con su ansiedad respecto al bienestar personal y social^(24)^. La bibliografía destaca los efectos del cambio climático en la salud de las mujeres, indicando que las personas que tienen una alta conciencia del cambio climático y de los acontecimientos medioambientales presentan niveles más elevados de ansiedad, preocupación y estrés, y que una mayor concienciación puede provocar ansiedad en las mujeres y afectar a su bienestar psicológico y a su salud mental, tanto de forma directa como indirecta^(15,26)^. Todos estos resultados de la investigación sugieren que los niveles de ansiedad de las mujeres aumentan debido a su alto nivel de concienciación sobre las consecuencias negativas del cambio climático.

Este estudio encontró que seguir las noticias sobre el cambio climático tenía un efecto significativo en la subescala de salud fisiológica (Figura 2). En particular, el árbol de decisión para la salud fisiológica (Figura 2) indica que tanto la conciencia sobre el cambio climático como el consumo de medios de comunicación afectan significativamente a esta subescala. Demir et al.^(24)^ informaron de que la mayoría de las mujeres de su estudio seguían las noticias sobre el cambio climático y obtenían la mayor parte de la información de fuentes como la televisión y las redes sociales/Internet^(24)^. Los estudios muestran que las personas acceden principalmente a las noticias sobre el cambio climático a través de medios digitales como la televisión y las redes sociales/Internet^(15,27)^. Maran y Begoti^(28)^ informaron de que las mujeres siguen la información sobre el cambio climático varias veces a la semana y que sus fuentes de información son principalmente Facebook, periódicos impresos y en línea, noticias y programas de televisión, y círculos familiares y de amigos. Los resultados de este estudio mostraron que las personas conocían el cambio climático y seguían las noticias en diferentes plataformas, lo que puede afectar a su nivel de ansiedad sobre el cambio climático.

El análisis CHAID de este estudio mostró que el conocimiento sobre el cambio climático era una de las variables que afectaban a la Escala de Ansiedad por el Cambio Climático para la Salud de la Mujer y a las subescalas de salud fisiológica y comportamiento (Figura 1–3). En el ámbito de las percepciones y opiniones de las participantes en el estudio de Korkmaz y Şahin, se detectaron diferencias significativas (*P* < 0,05) en cuanto a la conciencia de los efectos del cambio climático en sus vidas y el nivel de conocimiento sobre el cambio climático^(25)^. La dimensión conductual (Figura 3) también refleja que las personas informadas sobre el cambio climático son más propensas a adoptar comportamientos protectores, lo que coincide con los resultados de Stone et al.^(29)^, que relacionaron la concienciación con estrategias proactivas de salud mental y física en las mujeres. Süğüt y Vurgeç^(19)^ informaron de que, cuando se preguntó a las mujeres si sabían algo sobre el «cambio climático», el 39,8% respondió que sí y el 39,7% que lo sabía parcialmente. El conocimiento sobre el cambio climático depende en realidad de cómo las mujeres perciben la información y cómo la afrontan. Estas razones pueden variar en función de los niveles de ansiedad de las mujeres.

La Figura 4, que se centra en la dimensión específica de género de la ansiedad por el cambio climático, subraya la mayor carga psicológica que experimentan las mujeres. Este hallazgo coincide con los informes de la OMS y el ACOG, que destacan la interseccionalidad entre el género y los factores de estrés ambiental, especialmente entre las poblaciones vulnerables^(4,5)^. En conjunto, estos resultados sugieren que los niveles de ansiedad de las mujeres relacionados con el cambio climático están determinados por una compleja interacción de factores, entre los que se incluyen la concienciación, el acceso a la información y las percepciones individuales, tal y como respalda un creciente número de publicaciones internacionales.

Aunque ha aumentado el interés académico mundial por la intersección entre el cambio climático y la salud mental, siguen siendo escasos los estudios empíricos que se centran específicamente en la ansiedad relacionada con el cambio climático entre las mujeres en edad reproductiva, especialmente en el contexto de países de ingresos bajos y medios como Turquía. Además, los roles de género arraigados en la cultura y las expectativas sociales en materia de responsabilidad medioambiental pueden influir de manera única en las respuestas cognitivas y emocionales de las mujeres ante las amenazas relacionadas con el clima. Para dilucidar los mecanismos subyacentes y establecer relaciones causales, los estudios futuros deberían considerar el uso de diseños longitudinales y la incorporación de biomarcadores objetivos del estrés y los resultados en materia de salud.

### Puntos Fuertes y Limitaciones del Estudio

Este estudio presenta varios puntos fuertes dignos de mención. En primer lugar, contribuye a un área relativamente poco explorada al centrarse en la ansiedad por el cambio climático entre las mujeres en edad reproductiva en Turquía, abordando así una importante laguna en la bibliografía existente. Además, la utilización del análisis CHAID, un método sofisticado y ampliamente aceptado de árbol de decisión, permitió un examen en profundidad de las relaciones multifacéticas entre las variables y facilitó la identificación de patrones de interacción complejos que influyen en los niveles de ansiedad.

No obstante, el estudio no está exento de limitaciones. El diseño transversal restringe la capacidad de inferir relaciones causales entre las variables. Además, la recolección de datos se limitó a un único centro de salud familiar situado en Mersin, lo que puede limitar la validez externa y la generalización de los resultados a poblaciones más amplias.

## CONCLUSIÓN

Se ha constatado que conocer el cambio climático, seguir las noticias sobre el cambio climático y experimentar ansiedad por las consecuencias negativas del cambio climático son los principales factores que influyen en el nivel de ansiedad sobre el cambio climático en las mujeres en edad reproductiva. Conocer los efectos del cambio climático, que es uno de los problemas importantes de la actualidad, y determinar los factores subyacentes que lo afectan es muy importante para eliminar las preocupaciones sobre esta cuestión y adoptar medidas. Invertir en la salud de las mujeres es invertir en la salud de las generaciones actuales y futuras. Se debe apoyar el desarrollo de políticas y acciones a nivel nacional e internacional para minimizar las preocupaciones sobre el cambio climático. Durante la prestación de los servicios de atención, los profesionales de la salud deben proporcionar información sobre los efectos del cambio climático en la salud.

## Data Availability

Los conjuntos de datos utilizados y analizados durante el estudio están disponibles a pedido razonable del autor correspondiente.
